# Effect of Anti-TNF Antibodies on Clinical Response in Rheumatoid Arthritis Patients: A Meta-Analysis

**DOI:** 10.1155/2016/7185708

**Published:** 2016-07-31

**Authors:** Chunxiao Wu, Shengxu Wang, Peifeng Xian, Lu Yang, Ying Chen, Xianjie Mo

**Affiliations:** ^1^School of Traditional Chinese Medicine, Southern Medical University, Guangzhou, Guangdong 510515, China; ^2^Traditional Chinese Medicine-Integrated Hospital, The Affiliated Hospital of Southern Medical University, Guangzhou, Guangdong 510315, China

## Abstract

*Background*. Antitumor necrosis factor (anti-TNF) drugs have been applied for rheumatoid arthritis (RA) treatment; however, patients having anti-drug antibodies (ADAbs) do not benefit from these drugs. The meta-analysis aims to comprehensively assess the relationship between ADAb positive (ADAb+) and anti-TNF response in RA patients.* Methods*. Observational studies comparing different clinical response between ADAb+ and ADAb negative groups were included. Odds ratio (OR) with its corresponding 95% confidence interval (CI) was used as effect size. Subgroup analyses stratified by TNF inhibitor types and assay methods for ADAb detection were performed.* Results*. Totally, 10 eligible studies containing 1806 subjects were included. ADAb+ was significantly associated with reduced anti-TNF response to RA at all the time points after follow-up (*P* < 0.001). Subgroup analysis also supported this significant association (*P* < 0.05), except for enzyme-linked immunosorbent assay (ELISA) group at 3 months, infliximab (INF) and enzyme-linked immunosorbent assay (ELISA) groups at 6 months, and Immunological Multi-Parameter Chip Technology (IMPACT) group at 12 months.* Conclusion*. ADAb+ was significantly associated with reduced clinical response in RA patients, and other alternatives should be considered in RA patients presenting ADAb+.

## 1. Introduction

Rheumatoid arthritis (RA) is one of the autoimmune and chronic diseases [1]. Characterized by inflammation and destruction of joints, RA has caused increased mortality and substantial economic burden on patients worldwide [2].

Biologic therapies that target a specific inflammatory pathway or immune-related system could improve outcomes of RA patients, contributing to reduced mortality and comorbidity [3]. The* tumor necrosis factor* (*TNF*) encoded protein is a proinflammatory cytokine that is generated by macrophages in response to endotoxin [4]. Anti-TNF agents were firstly confirmed as the biologic drugs for RA treatment when nonbiologic disease modified antirheumatic drugs (DMARDs) were failing [5]. At present, five anti-TNF drugs have been licensed for the clinical use of RA, including infliximab (INF), adalimumab (ADAL), etanercept (ETN), golimumab (GLM), and certolizumab (CTZ) [6–9]. However, since TNF has significant roles in host defense, numerous studies find that anti-TNF application is also related to increased risk of serious infections and malignancies in RA patients [10–12]. Moreover, several patients who generate the anti-drug antibodies (ADAbs) do not benefit from the anti-TNF drugs [13].

Previously, several meta-analyses have been carried out to evaluate the safety of anti-TNF agents. For instance, Bongartz et al. discovered that two anti-TNF agents, INF and ADAL, could result in increased risk of malignancies in RA patients in a dose-dependent manner [6]. Another meta-analysis indicates anti-TNF-*α* is correlated with decreased risk of all cardiovascular events [14]. Aaltonen et al. compare the efficacy and safety of anti-TNF drugs with methotrexate and find that there are no significant differences between them in efficacy, while ETN has a reduced risk of adverse event and is proposed as the safest alternative [15]. However, causative factors for these results are not considered, nor the drug immunogenicity or ADAb. In addition, different time points in these studies might produce different results.

Therefore, we performed this meta-analysis, mainly concerning the ADAb status (ADAb positive or negative), to comprehensively assess association between ADAb+ and response to anti-TFN agents, aiming to give a precise assessment of application of anti-TNF for RA management.

## 2. Methods

### 2.1. Search Strategy

Literature search was conducted in databases such as PubMed, Embase, and Cochrane Library. The search strategy was (“Immunogenicity” OR “response”) AND (“rheumatoid arthritis” OR “RA”) AND (“anti-tumor necrosis factor” OR “anti-TNF” OR “TNF-*α* antagonist” OR “TNF inhibitors” OR “infliximab” OR “adalimumab” OR “etanercept” OR “golimumab” OR “certolizumab” OR “infliximab biosimilar”) AND (“antibody” OR “ADAb”). There was no language restriction and the searching was set before January 5, 2016. Additionally, manual search for studies that were published in paper was conducted. Reference lists of included studies were also scanned for more eligible studies.

### 2.2. Inclusion and Exclusion Criteria

The inclusion criteria were as follows: (1) subjects were RA patients ≥18 years old; (2) chemotherapies were anti-TNF agents such as INF, ADAL, ETN, GLM, and CTZ; (3) the studies compared therapeutic differences between ADAb positive (ADAb+) and ADAb negative (ADAb−) RA patients; (4) the outcome was clinical response with the measurement criteria of the American College of Rheumatology (ACR) or European League Against Rheumatism (EULAR) criteria for RA; (5) the study type was observational study.

The exclusion criteria were as follows: (1) no control group was contained in the study; (2) data were incomplete or the results could not be used for statistical analysis; (3) the studies were reviews, letters, or comments.

### 2.3. Data Extraction

Two investigators independently completed the literature selection based on the predefined criteria. Then, the following required data were abstracted, such as first-author name, publication year, study region, TNF types, subjects' characteristics (e.g., sample size, age, gender composition, and course of disease), time point in observational studies, sample sizes, and case numbers of outcomes in ADAb+ group and ADAb− group, respectively. Disagreements were resolved through discussion with a third investigator.

### 2.4. Statistical Analysis

Odds ratio (OR) with its corresponding 95% confidence interval (CI) was used as a measure of the effect size to calculate differences of clinical response between ADAb+ and ADAb− groups. Heterogeneities across studies were determined by Cochrane's *Q* statistic and *I* ^2^ test [16]. If extensive heterogeneity was detected (*P* < 0.05, *I* ^2^ > 50%), a randomized-effects model was applied, whereas if there was no obvious heterogeneity (*P* > 0.05, *I* ^2^ ≤ 50%), a fixed-effects model was applied.

Outcomes were pooled at different time points (3 months, 6 months, 12 months, and ≥24 months) after follow-up, respectively. Subgroup analyses stratified by TNF inhibitor (TNFi) type and assay method for ADAb detection were performed to further explore influences of these specific factors on the outcomes. Publication bias was determined using Egger's test [17]. The Stata11.0 software (STATA, College Station, TX, USA) was used to complete all the statistical analyses.

## 3. Results

### 3.1. Eligible Studies

**Figure 1:**
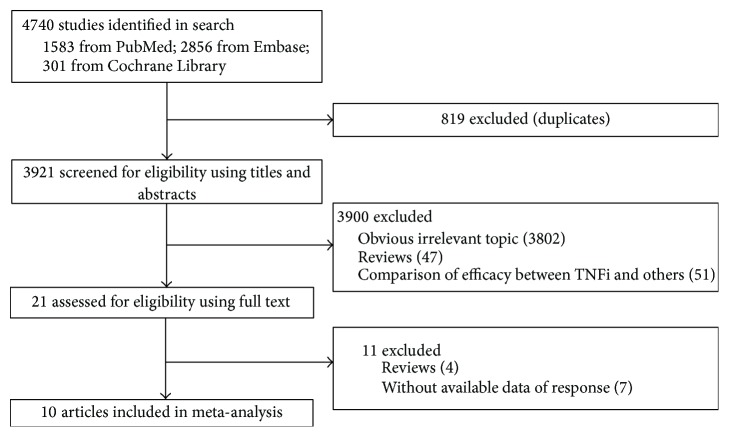
Procedures of study selection.

Specific procedures of the study selection are listed in Figure 1. By the preliminary search, a total of 4740 studies were retrieved, and 3921 remained after eliminating duplicated publications. Through title and abstract screening, another 3900 studies (including 3802 studies that obviously did not conform to the inclusion criteria, 47 reviews, and 51 studies that compared the therapeutic effects of TNFi with other drugs on RA) were excluded. Eleven out of the remaining 21 studies were removed after full-text reading. No additional studies were added via manual search. As a result, 10 eligible studies [18–27] were included in the meta-analysis.

### 3.2. Characteristics of the Included Studies

As indicated in Table 1, the 10 studies consisted of a total of 1806 subjects. These studies were published from 2007 to 2015, and most of them were conducted in European countries except Chen et al.'s study [20], which was carried out in China. Three ADAb detection methods, radioimmunoassay (RIA), enzyme-linked immunosorbent assay (ELISA), and Immunological Multi-Parameter Chip Technology (IMPACT), and 6 time points of the outcomes, including 3, 6, 12, 24, 36, and 48 months after follow-up, were involved.

### 3.3. Meta-Analysis and Subgroup Analysis

**Figure 2:**
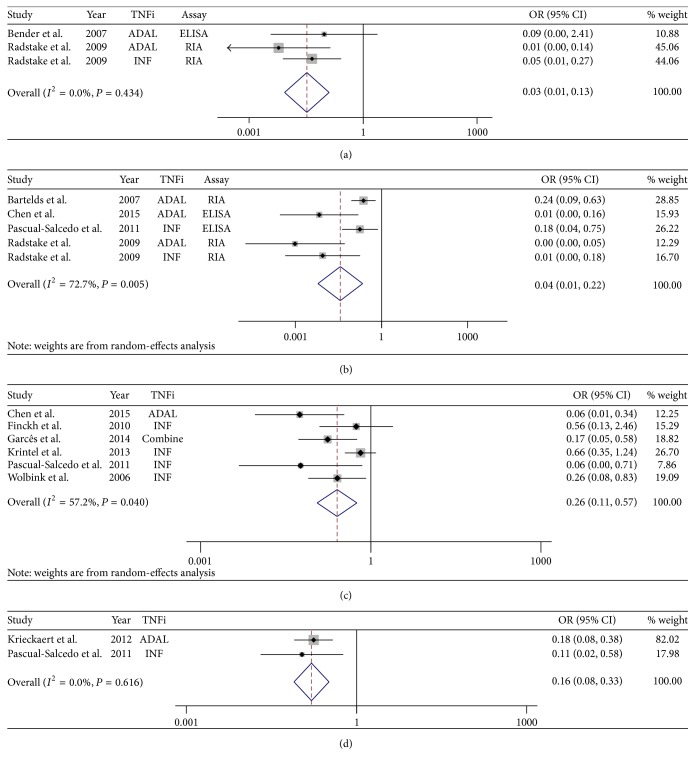
Effect of ADAb+ on anti-TNF response in rheumatoid arthritis patients, compared with ADAb− group at different time points after follow-up. (a) At 3 months; (b) at 6 months; (c) at 12 months; (d) at >24 months.

A fixed-effects model was used for comparisons at the time points of 3, 12, 24, 36, and 48 months after follow-up, due to the lack of significant heterogeneity (*P* > 0.05, *I* ^2^ ≤ 50%). By contrast, a randomized-effects model was applied 6 months after follow-up due to significant heterogeneity (*I* ^2^ = 72.7%, *P* = 0.005). As expected, ADAb+ was significantly associated with reduced anti-TNF response to RA at all the time points after follow-up (3 months: OR = 0.03, 95% CI: 0.01 to 0.13, *P* < 0.001; 6 months: OR = 0.04, 95% CI: 0.01 to 0.22, *P* < 0.001; 12 months: OR = 0.26, 95% CI: 0.11 to 0.57, *P* < 0.001; ≥24 months: OR = 0.16, 95% CI: 0.08 to 0.33, *P* < 0.001) (Figure 2).

Subgroup analysis stratified by TNFi types and assay methods also supported this significant association (*P* < 0.05), except for ELISA group at 3 months (OR = 0.10, 95% CI, 0.01 to 2.41), INF (OR = 0.05, 95% CI, 0.00 to 1.06) and ELISA (OR = 0.05, 95% CI, 0.00 to 1.13) groups at 6 months, and IMPACT group at 12 months (OR = 0.66, 95% CI, 0.35 to 1.24) (Table 2).

### 3.4. Publication Bias

As only a few studies compared the clinical response between the two groups at 3, 6, and ≥24 months after follow-up, we just evaluated the publication bias at the time point of 12 months. Egger's test indicated that there lacked significant publication bias (*P* = 0.067).

## 4. Discussion

In the present study, a total of 10 studies were included involving 1806 subjects. Meta-analysis indicated that ADAb+ group had significant association with reduced anti-TNF response (*P* < 0.05), whatever the time point was. Moreover, in most of the subgroups stratified by TNFi types and assay methods, significant association was also detected between ADAb+ group and decreased anti-TNF response, except in ELISA group at 3 months, INF and ELISA groups at 6 months, and IMPACT group at 12 months. Studies that used RIA assay all confirmed this significant association at each time point.

Five anti-TNF drugs have been approved for RA treatment. A large number of randomized clinical trials and observational studies have confirmed the effectiveness of anti-TNF agents for RA treatment, whether at an early stage or during a long period [28–30]. However, multiple studies point out that the RA patient prescribed these drugs is at an increased risk of serious infections and malignancies [31–33]. In addition, in several patients, anti-TNF agents are ineffective for RA management [34]. ADAb generated in patients is one causative factor for nonresponse to anti-TNF drugs such as INF and ADAL for RA treatment [35, 36]. Reportedly, more INF doses are needed in RA patients with anti-INF antibodies, and high ADAb level of anti-INF is related to the loss of clinical response, which would discount the therapeutic efficacy [25]. Several studies indicate that different bioavailability and diverse development of ADAb in RA patients may be the major reasons for different clinical responses [37, 38].

Immune response to anti-TNF therapy contributes to the generation of ADAbs, which could impair the clinical response if they are able to decrease the serum levels of the active drug [39]. Immunogenicity of the anti-TNF agents is considered as the main mechanism of treatment failure [40]. The immunogenicity rate of different anti-TNF drugs is different, due to diversities in various factors such as administration route, drug dose, concomitant medication, detailed treatment schedule, and immune and nutritional status [41]. Extensive studies have investigated influence of the immunogenicity of anti-TNF agents on drug efficacy and safety and found that antibodies of these agents, such as INF, could affect the drug's pharmacokinetics, which may lead to delayed infusion and injection site reactions [41, 42]. Meanwhile, the anti-chimeric antibodies (ATIs), induced by INF, could increase the clearance of INF and attenuate its function [43]. This might be the reasonable explanation for our finding of the significant association between ADAb+ and reduced response rate.

On the other hand, the detection of ADAb might be influenced by different collection times and methods. For instance, ATIs may be undetectable at the initiation of INF administration due to the fact that they generate the immune complexes with the drug [41]. With regard to the detection methods, a bridging ELISA is the most common assay for ADAb detection [34]; however, it could be influenced by a high rate of false positive results [44]. A study indicates that an underestimated ADAb+ rate might be due to undetectable ADAb using the bridging ELISA [45]. Therefore, it is understandable that, in ELISA group at 3 months and 6 months, there was not any observed association between ADAb+ and reduced response, which may be attributed to the reduced detection rate by ELISA method.

IMPACT is a multiplex platform that is a novel technology for ADAb detection, and, reportedly, it has greater sensitivity for ADAb detection, compared with other current clinical tests [46]. However, the duration in this study is as short as <6 months. Another study points out that IMPACT is a novel method mainly used for definition of clinical stages of RA at molecular level [47]. Therefore, whether this method is precise and sensitive in a long duration needs to be further investigated. Our discovery that, in IMPACT group at 12 months, ADAb+ was not associated with decreased clinical response provides a hint that IMPACT might not be sensitive for ADAb detection at a long duration. However, it needs to be verified by more studies. For RIA assay, a previous study that assessed INF levels and ADAbs for a long duration (1.5 to 18 months) using different methods found that fluid-phase RIA was better than cross-binding ELISA [48]. Therefore, it is understandable that whatever the time point, ADAb was significantly associated with reduced anti-TNFi clinical response using this detection method.

Our study has the following advantages: (1) the clinical response was pooled at different time points, respectively, which avoided potential bias generated from drug effect varied by different time points; (2) subgroup analyses stratified by TNFi types and assay methods for ADAb detection were performed, contributing to exploration of more precise correlations between ADAb+ and response to specific anti-TNF drugs in RA patients; (3) no obvious publication bias was observed in this meta-analysis, indicating the reliability of our results. However, there were also several limitations as follows: (1) all the included studies were observational studies, and the confounding factors could not be well controlled; (2) significant heterogeneity was detected at several time points, which might cause deviation to some extent; (3) drug level in different studies might be a confounding factor that influences the result; however, it was not taken into consideration in our study; (4) the sample size was relatively small, and large-scale studies with more samples are required to confirm our findings.

In conclusion, ADAb+ was significantly associated with reduced clinical response in RA patients, and other alternatives should be considered in RA patients presenting ADAb+.

## Figures and Tables

**Table 1:** Characteristics of the included studies in this meta-analysis.

Study	Year	Area	TNFi	*N* (%, female)	Age	Disease duration (ys)	MTX (%)	PRED (%)	Assay	Follow-up (months)	ADAb+	Outcome (*n*)	ADAb−	Outcome (*n*)
Bartelds et al. [18]	2007	Netherlands	ADAL	121 (79)	53 ± 13	12 ± 10	79	34	RIA	6	21	9	100	76
Bender et al. [19]	2007	Germany	ADAL	15 (66.7)	55.9 ± 8.1	12.2 ± 8.2	66.7	100	ELISA	3	13	4	2	2
Chen et al. [20]	2015	Taiwan	ADAL	36 (88.9)	52.9 ± 15.0	5.44 ± 2.42	88.9	NA	ELISA	6	8	2	28	28
									ELISA	12	10	3	26	23
									RIA	12	13	6	23	20

Finckh et al. [21]	2010	Switzerland	INF	64 (76.6)	59.3 (NA)	13.8 (9.7)	81.3	46.9	ELISA	12	8	4	56	36
Garcês et al. [22]	2014	Portugal	Combine	105 (87)	54 ± 14	10.1 (4.6–12.6)^*∗*^	97	98	ELISA	12	18	4	87	54
Krieckaert et al. [23]	2012	Netherlands	ADAL	204 (76)	54 ± 12	7 (3–16)^*∗*^	77	32	RIA	36	54	9	150	80
Krintel et al. [24]	2013	Denmark	INF	218 (80)	56 (21–86)	6 (0–56)	91	24	IMPACT	12	79	27	84	37
Pascual-Salcedo et al. [25]	2011	Finland	INF	85 (81.0)	53.8 ± 14.2	NA	81	74	ELISA	6	16	9	33	29
										12	7	4	24	23
										48	11	6	36	33
Radstake et al. [26]	2009	Netherlands	INF	35 (86.0)	57 ± 10	NA	100	29	RIA	3	14	3	21	18
										6	18	4	17	17
			ADAL	34 (79.0)	56 ± 10	NA	41	26	RIA	3	8	0	26	24
										6	10	0	24	24
Wolbink et al. [27]	2006	Netherlands	INF	51 (82.4)	56 ± 13	12 ± 9	86	0	ELISA	12	22	8	29	20

TNFi: tumor necrosis factor inhibitors; MTX: methotrexate; ADAL: adalimumab; INF: infliximab; ETN: etanercept; combine: INF/ETN/ADAL; PRED: prednisone; ^*∗*^median (IQR, interquartile range); NA: not available; ELISA: enzyme-linked immunosorbent assay; RIA: radioimmunoassay; IMPACT: Immunological Multi-Parameter Chip Technology; ADAb+: anti-drug antibodies positive; ADAb−: anti-drug antibodies negative; outcome: good+ moderate EULAR responders.

**Table 2:** Subgroup analyses stratified by TNFi types and assay methods for ADAb detection.

Group or subgroup	3 months			6 months			12 months			≥24 months		
	*n*	Model	OR (95% CI)	*n*	Model	OR (95% CI)	*n*	Model	OR (95% CI)	*n*	Model	OR (95% CI)
Total	3	F	0.03 (0.01, 0.13)	5	R	0.04 (0.01, 0.22)	6	R	0.26 (0.11, 0.57)	2	F	0.16 (0.08, 0.33)

*TNFi*												
ADAL	2	F	0.02 (0.00, 0.21)	3	R	0.02 (0.00, 0.62)	1	—	0.06 (0.01, 0.34)	1	—	0.18 (0.08, 0.38)
INF	1	—	0.05 (0.01, 0.27)	2	R	0.05 (0.00, 1.06)	4	F	0.49 (0.29, 0.81)	1	—	0.11 (0.02, 0.58)
Combine	NA	NA	NA	NA	NA	NA	1	—	0.18 (0.05, 0.58)	NA	NA	NA

*Assay*												
RIA	2	F	0.03 (0.01, 0.11)	3	R	0.02 (0.00, 0.63)	NA	NA	NA	1	—	0.18 (0.08, 0.38)
ELISA	1	—	0.10 (0.01, 2.41)	2	R	0.05 (0.00, 1.13)	5	F	0.20 (0.10, 0.38)	1	—	0.11 (0.02, 0.58)
IMPACT	NA	NA	NA	NA	NA	NA	1	—	0.66 (0.35, 1.24)	NA	NA	NA

TNFi: tumor necrosis factor inhibitors; ADAL: adalimumab; INF: infliximab; ETN: etanercept; combine: INF/ETN/ADAL; OR: odds ratio; CI: confidence interval; R: randomized-effects model; F: fixed-effects model; —: no model was used; NA: not available; ELISA: enzyme-linked immunosorbent assay; RIA: radioimmunoassay; IMPACT: Immunological Multi-Parameter Chip Technology.

## Abbreviations

TNF: Tumor necrosis factor
ADAb+: Anti-drug antibody positive
ADAb−: Anti-drug antibody negative
OR: Odds ratio
CI: Confidence interval
TNFi: TNF inhibitor
INF: Infliximab
ADAL: Adalimumab
ETN: Etanercept
Combine: INF/ETN/ADAL
ELISA: Enzyme-linked immunosorbent assay
RIA: Radioimmunoassay
IMPACT: Immunological Multi-Parameter Chip Technology.
